# Management of cancer-associated venous thromboembolism: Perspectives on optimizing current therapeutics with a focus on factor XI inhibition

**DOI:** 10.1007/s11239-025-03154-7

**Published:** 2025-07-29

**Authors:** Massimiliano Camilli, Giovanni Occhipinti, Nicola Potere, Claudio Laudani, Xavier Freixa, Antonella Lombardo, Francesco Burzotta, Marcello Di Nisio, Marc Carrier, Teresa Lopez-Fernandez, Nicola Maurea, Bianca Rocca

**Affiliations:** 1 Department of Cardiovascular and Pulmonary Sciences, Catholic University of the Sacred Heart, Rome, Italy; 2 Department of Cardiovascular Medicine, Fondazione Policlinico Universitario A. Gemelli IRCCS, L.go A. Gemelli, 1, Rome, 00168 Italy; 3 Cardiovascular Clinic Institute, Institut d’Investigacions Biomèdiques August Pi i Sunyer (IDIBAPS), Hospital Clínic, University of Barcelona, Barcelona, Spain; 4 Facultat de Medicina I Ciències de la Salud, Universitat de Barcelona (UB), Barcelona, Spain; 5 Department of Medicine and Ageing Sciences, “G. d’Annunzio” University, Chieti, Italy; 6 Division of Cardiology, Azienda Ospedaliero-Universitaria Policlinico “Rodolico - San Marco”, University of Catania, Catania, Italy; 7 Department of Medicine, University of Ottawa and Ottawa Hospital Research Institute, Ottawa, ON Canada; 8 Cardio-Oncology Unit, Cardiology Department, La Paz University Hospital, IdiPAZ Research Institute, Madrid, Spain; 9 Cardio-Oncology Unit, Cardiology Department, Quironsalud University Hospital, Madrid, Spain; 10 Division of Cardiology, Istituto Nazionale Tumori-IRCCS-Fondazione G. Pascale, Napoli, Italy; 11 Department of Medicine and Surgery, LUM University, Casamassima, Bari, Italy

**Keywords:** Cardio-oncology, Venous thromboembolism, Cancer-associated thrombosis, Novel anticoagulants, Factor XI inhibitors

## Abstract

Cancer-associated thrombosis (CAT) encompasses manifestations of deep venous thrombosis and/or pulmonary embolism occurring during the evolution of cancer. CAT represents one of the major cardiovascular complications associated with cancer and anti-cancer treatments, and the second leading cause of death after cancer progression. The rate of venous thromboembolism (VTE) recurrence is augmented in patients with cancer, together with the risk of bleeding, when compared with subjects without malignancy. Thus, decisions on optimal anticoagulation strategy should carefully balance both thrombotic and bleeding risk. While low-molecular weight heparins and direct oral anticoagulants now represent the standard-of-care in patients with cancer, newer pharmacologic compounds able to prevent VTE recurrence while minimizing the hemorrhagic risk are needed, and currently under investigation. In particular, factor XI inhibitors have emerged as potentially safe drugs in this highly vulnerable population, although results from dedicated clinical trials are waited to confirm this hypothesis. This review aims to summarize current management, controversies, and latest developments in pharmacotherapeutic approaches for patients with CAT.

**Supplementary Information:**

The online version contains supplementary material available at 10.1007/s11239-025-03154-7.

## Graphical abstract

**Figure:**
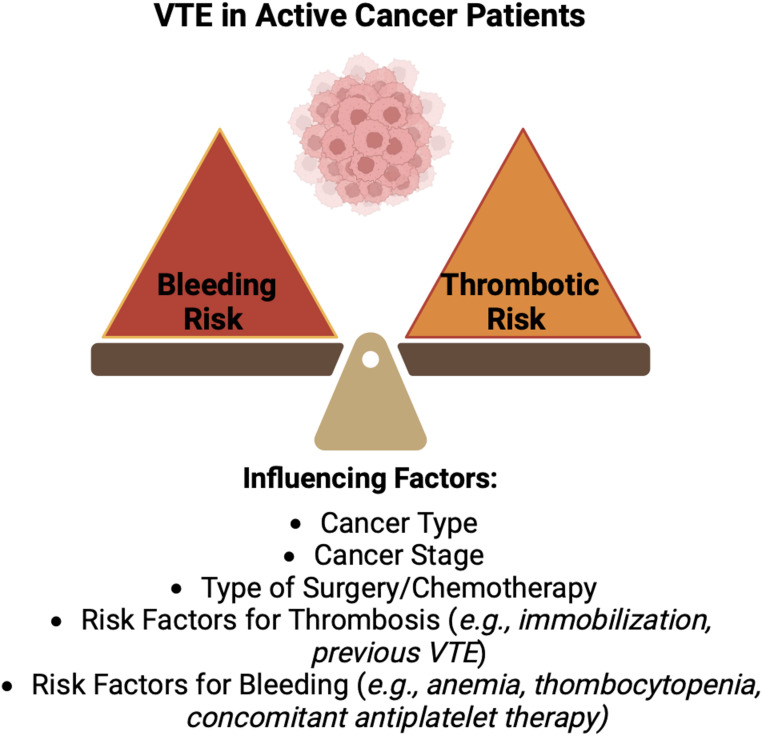
The choice of the anticoagulation strategy in patients with cancer should be carefully balanced between the risk of recurrent thromboembolism and that of bleeding. Abbreviations: VTE, venous thromboembolism

## Cancer-associated thromboembolism: definition, epidemiology and risk factors

Venous thromboembolism (VTE) includes pulmonary embolism (PE) and deep venous thrombosis (DVT). VTE is a multifactorial disease: its occurrence depends on a constellation of risk factors and known predisposing conditions, which may impact differently in the pathophysiology and evolution of the thrombotic process, also because of their transient or persistent nature [1, 2]. The strength of different risk factors for VTE supports the classification in: provoked, minimally provoked, or unprovoked disease [1, 2].

**Fig. 1:**
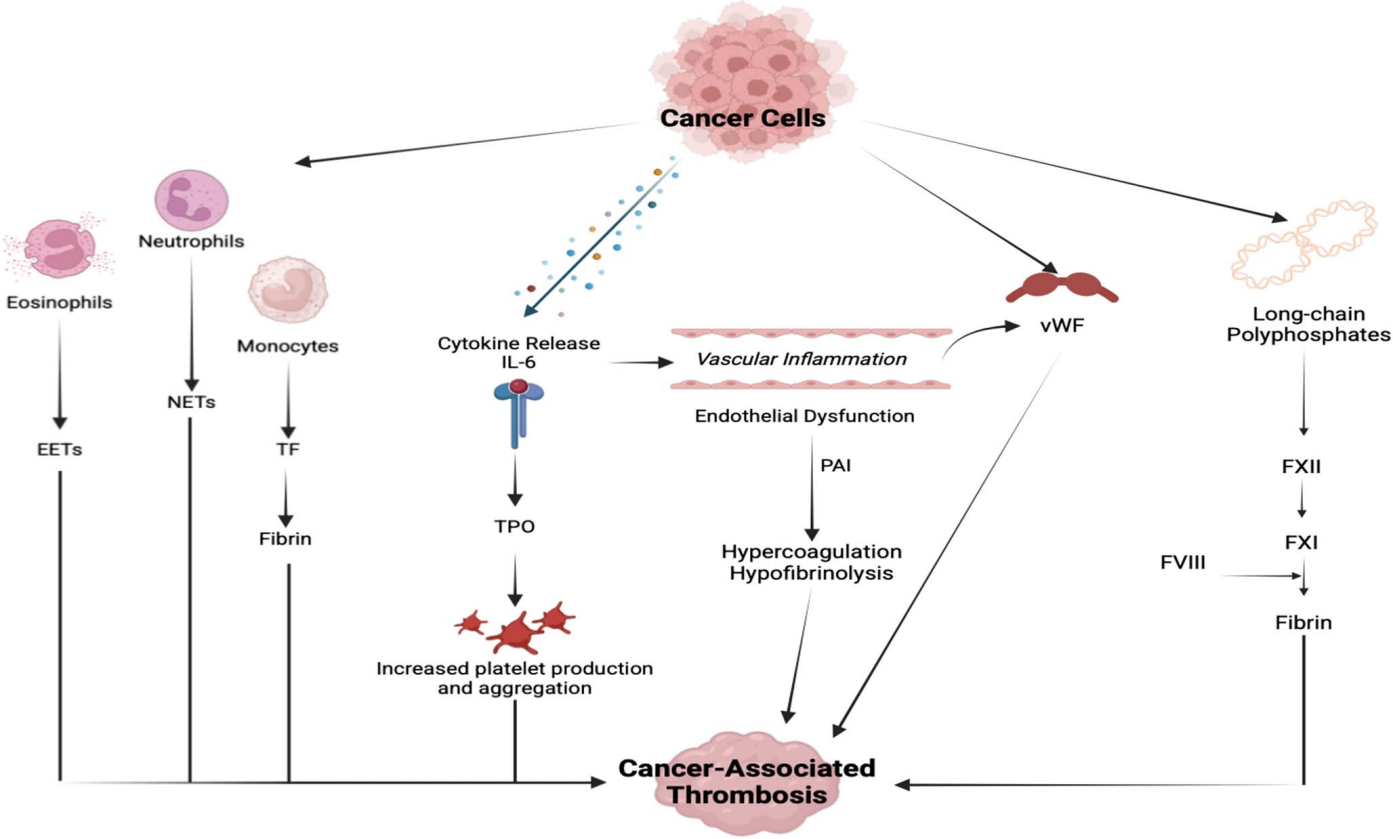
Main mechanisms involved in cancer-associated thrombosis (CAT). Cancer cells stimulate leucocytes, in particular neutrophils that generated neutrophil extra-cellular traps (NETs), that in turn enhance the thrombotic process. Tumor cells also induce tissue factor (TF) expression on monocytes, initiating blood coagulation, although certain types of cancer cells also express TF on their cell surface, and directly trigger blood coagulation. Eosinophils may contribute to thrombus formation through eosinophil extracellular traps (EETs) as well. Cytokine release induced by tumor cells, in particular interleukin-6 (IL-6) production, stimulates hepatic thrombopoietin (TPO) synthesis, leading to platelet production and thrombosis enhancement. At the same time, tumor-derived plasminogen activator inhibitor (PAI), that may also be released in case of endothelial dysfunction, inhibits plasminogen activators (including tissue plasminogen activator and urokinase-type plasminogen activator), therefore reducing the generation of plasmin and resulting in hypofibrinolysis. Endothelial dysfunction induces release of von Willebrand factor (vWF), with subsequent platelet adhesion and activation, and thrombus formation. Lastly, certain types of tumors produce long-chain polyphosphates which can activate factor XII (FXII). Activated FXII further triggers factor XI (FXI) and the intrinsic coagulation pathway in the presence of activated factor VIII (FVIII), perpetuating the thrombotic process. Abbreviations: EETs, eosinophil extracellular traps; FVIII, factor VIII; FXI, factor XI; FXII, factor XII; IL-6, Interleukin-6; PAI, plasminogen activator inhibitor; TF, tissue factor; TPO, thrombopoietin; vWF, von Willebrand factor

Active cancer introduces a major provoking factor for VTE, especially in patients receiving chemotherapy [1, 2]. The term cancer-associated thrombosis (CAT) encompasses manifestations of VTE occurring in the context of cancer [1, 2]. VTE represents one of the major causes of morbidity and mortality in cancer patients [3, 4]. The risk of VTE is 4-to-12-fold higher in cancer patients compared to the general population [5–7], with varying incidences according to the type of cancer (the highest risk is for stomach, pancreatic, ovarian and lung cancer, together with multiple myeloma), as well as the stage of cancer (metastatic/advanced disease are at the highest risk) [5–9]. In advanced disease, tumors can directly compress veins, resulting in venous stasis and subsequent VTE. The molecular mechanisms involved in the CAT pathogenesis are shown in Fig. 1.

The thrombotic risk also varies according to patient-related risk factors, including age, prolonged immobilization, comorbidities and history of cardiovascular disease or cardiovascular risk factors (i.e., diabetes, hypertension, obesity, dyslipidemia) [6–8, 10]. Recently, the role of genetic predisposition, in particular of single nucleotide polymorphisms in coagulation-related genes (i.e., factor V Leiden and prothrombin G20210A), has emerged [11, 12]. At the same time, oncogenic mutations and rearrangements might also be involved, with a substantial increase in CAT risk (Table 1) [11, 13].

**Table 1:** Cancer genetics factors influencing the risk of venous thromboembolism and related to the tumor itself

Gene involved	Tumor expression	VTE incidence reported in clinical studies
ALK	Non-small cell lung cancer	26.9–47.1%
EGFR	Non-small cell lung cancer	9–35%
KRAS	Colorectal cancer Lung cancer	16.1–54%
JAK2	Hematopoietic malignancies/myeloproliferative neoplasm	12%
IDH1/IDH2	Brain tumors	18.2–25.6%
ROS1	Non-small cell lung cancer	34.6–41.6%

Refer to Reference 13 in the text

Pharmacologic compounds used to manage the cancer also play a role in VTE occurrence [8, 14] (see Table 2). Conventional chemotherapy, targeted therapies (including immunotherapies) and hormonal treatment are indeed responsible for enhanced thrombogenicity through several mechanisms, in particular endothelial dysfunction, increased platelet activation, immune cell activation and induction of inflammatory cytokines [15]. As well, recent major surgery or need for central venous catheters should be both considered as predisposing conditions to VTE [6–8].

**Table 2:** Pharmacological compounds used in patients with cancer that increase the risk of thrombosis

**Chemotherapy/anticancer therapy**	o Cisplatin-based chemotherapy o Antimetabolites: capecitabine, gemcitabine, paclitaxel o Taxanes o Irinotecan o Anthracyclines o Cyclophosphamide o Immunomodulatory drugs: thalidomide, lenalidomide o Proteasome inhibitors (in particular carfilzomib) o Aromatase inhibitors and Tamoxifen o Androgen-deprivation therapy o Anti-angiogenic agents (e.g., bevacizumab, ramucirumab, sunitinib, sorafenib, pazopanib, axitinib, cabozantinib, regorafenib, lenvatinib, vandetanib, aflibercept) o BCR-ABL tyrosine kinase inhibitors (nilotinib, ponatinib)(mainly involved in arterial thrombosis)
**Others**	o Erythropoiesis-stimulating agents o Platelet stimulating factors

Of importance, VTE is the second cause of death after cancer progression (> 60% within the first year following the VTE diagnosis) [6–8]. In addition, patients with cancer experience a high risk of VTE recurrence (up to 20% in the first 12 months after anticoagulation interruption) with a case fatality rate of almost 15%, while the risk of breakthrough thrombosis is considered as low as 7–8% during the first 6 months of anticoagulant treatment [6–8].

The rate of bleeding events is increased in this population, with a 12-month cumulative incidence rate of major bleeding of 12.4% (95% CI: 6.5–18.2), and a case fatality rate of 8.9% (95% CI: 3.5–21.1). The risk is highest for thrombocytopenic subjects, those affected by gastrointestinal, prostate cancer and hematologic malignancies, or for patients with intracranial lesions [8, 9].

Although VTE occurrence may be associated with acute manifestations, up to 50% of VTE events are incidentally detected during imaging performed for cancer staging, evaluation of treatment response and follow-up [11–14]. Interestingly, even if VTE diagnosis was not suspected in these subjects, approximately one-half of them reported symptoms of PE or DVT [11, 14–17]. However, as these symptoms are generally non-specific, they are often attributed to chemotherapy side-effects or cancer itself. Recent studies showed that incidental PE (as well as subsegmental PE) may carry a risk of VTE recurrence and mortality similar to symptomatic PE [16, 17]. Therefore, patients with active cancer and incidental PE should be managed as symptomatic cases [16, 17].

In order to mitigate both the risk of VTE recurrence and bleeding complications (**Graphical Abstract**), many efforts have been made in terms of VTE pharmacologic prevention and treatment. The aim of this review is to summarize the current knowledge and recommendations for the treatment of cancer-associated VTE; we will also shed light on residual challenges of anticoagulation, and explore latest advances on the role of factor XI inhibitors.

## Methods

A literature search was performed using MEDLINE for articles published between January 1, 2013, and February 1, 2025. Articles were selected only if published in English.

Literature search terms used for article selection are reported in Supplementary Material, Table 1. Articles were screened for eligibility by title and abstract. Articles were excluded if there was not at least one outcome variable of interest, samples did not include patients with cancer, and the article’s primary focus was not on the treatment or secondary prophylaxis of cancer-associated VTE. Additional key references from personal libraries and other sources were identified, and appraised by the authors for inclusion.

### Treatment of cancer-associated venous thromboembolism: Available evidence

Low molecular-weight heparins (LMWHs) and direct oral anticoagulants (DOACs) represent the cornerstone treatments of CAT in clinical practice [8, 11, 18–20]. Until 2002, Vitamin K antagonists (VKAs) were considered the standard of care for VTE pharmacological management, even in patients with active cancer [7, 8, 11, 18–20]. Thereafter, several clinical trials have attempted to compare VKAs with LMWHs, demonstrating that the latter compounds were superior or similar at preventing recurrent VTE, and led to similar major bleeding events, after which, LMWH became the standard therapy for CAT [7, 8, 11, 18–20]. Recent landmark trials have indeed showed that DOACs, in particular Factor Xa inhibitors, such as apixaban, rivaroxaban and edoxaban, are at least as effective as LMWH in preventing VTE recurrence, and associated with a non-significant increase in major bleeding events. In these studies, the common comparator was the same regimen of dalteparin, relying on the results of the CLOT trial [21], and the study treatment durations were 6–12 months [22–26]. At the same time, DOACs have been associated with overall higher risk for clinically relevant non-major bleeding events. Pooled analyses of data from clinical trials also showed substantial absolute incidence rates of major bleeding (almost 5% at 6 months) with DOACs, in particular in the subgroups of patients with unresected gastrointestinal or genitourinary cancers [26–29].

**Fig. 2:**
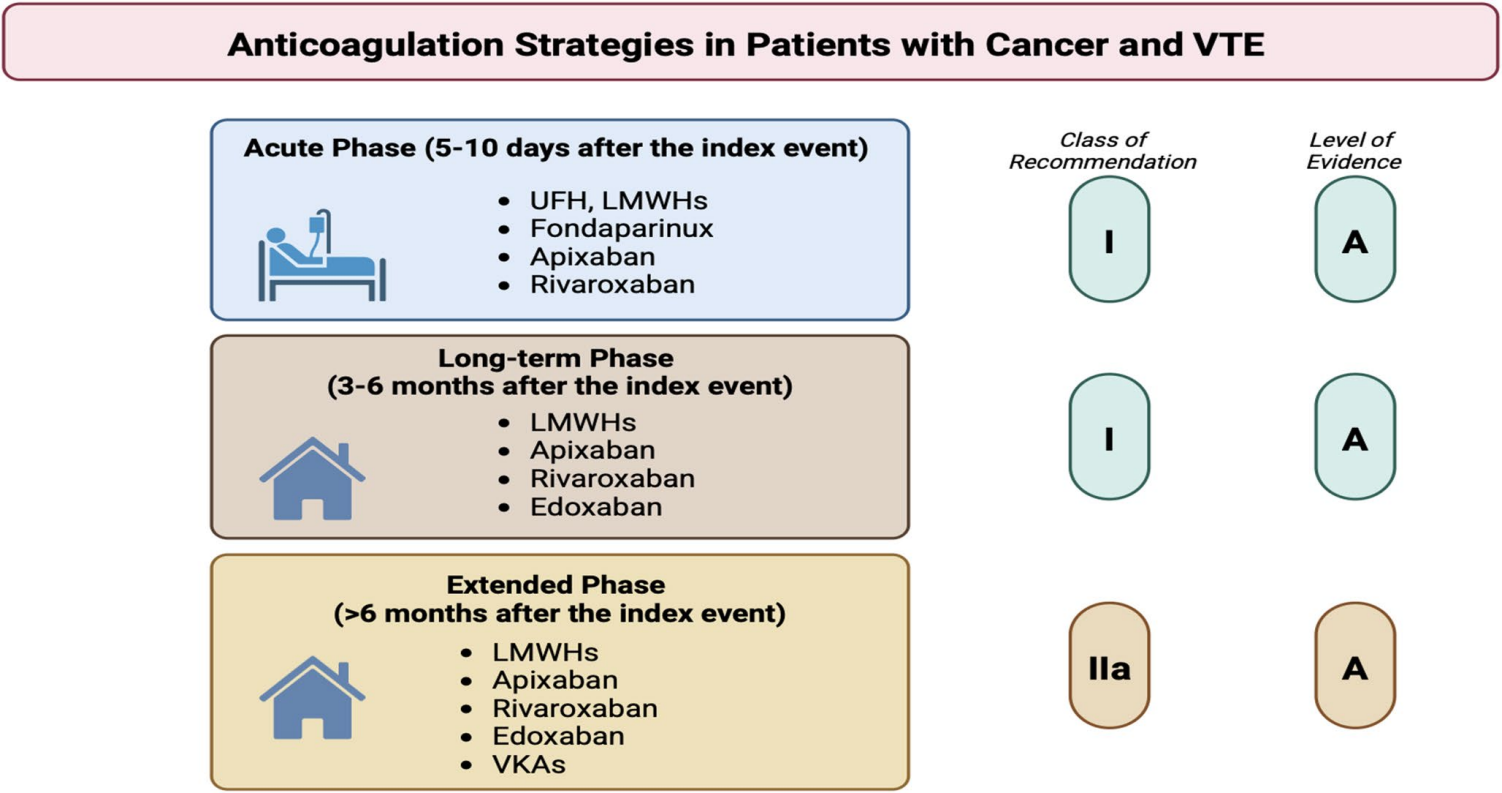
Current recommendations for venous thromboembolism (VTE) management in patients with cancer. Pharmacologic strategies for VTE treatment should be divided into three phases according to the temporal relationship with the index event. The acute phase, occurring in the first 5–10 days; the long-term phase lasts for the first 3–6 months after the index event, while the extended phase goes beyond the first 6 months from the index event. *Referred to Falanga A*,* Ay C*,* Di Nisio M*,* Gerotziafas G*,* Jara-Palomares L*,* Langer F*,* Lecumberri R*,* Mandala M*,* Maraveyas A*,* Pabinger I*,* Sinn M*,* Syrigos K*,* Young A*,* Jordan K; ESMO Guidelines Committee. Venous thromboembolism in cancer patients: ESMO Clinical Practice Guideline. Ann Oncol. 2023 May;34* [5]:*452–467. doi*: 10.1016/j.annonc.2022.12.014. *Epub 2023 Jan 10. PMID: 36,638,869; 8. Lyon AR*,* López-Fernández T*,* Couch LS*,* Asteggiano R*,* Aznar MC*,* Bergler-Klein J*,* Boriani G*,* Cardinale D*,* Cordoba R*,* Cosyns B*,* Cutter DJ*,* de Azambuja E*,* de Boer RA*,* Dent SF*,* Farmakis D*,* Gevaert SA*,* Gorog DA*,* Herrmann J*,* Lenihan D*,* Moslehi J*,* Moura B*,* Salinger SS*,* Stephens R*,* Suter TM*,* Szmit S*,* Tamargo J*,* Thavendiranathan P*,* Tocchetti CG*,* van der Meer P*,* van der Pal HJH; ESC Scientific Document Group. 2022 ESC Guidelines on cardio-oncology developed in collaboration with the European Hematology Association (EHA)*,* the European Society for Therapeutic Radiology and Oncology (ESTRO) and the International Cardio-Oncology Society (IC-OS). Eur Heart J. 2022 Nov 1;43* [41]:*4229–4361. doi*: 10.1093/eurheartj/ehac244. Abbreviations: LMWHs, low-molecular weight heparins; UFH, unfractioned heparin; VKAs, vitamin K antagonists; VTE, venous thromboembolism

Of importance, apixaban and rivaroxaban are the only DOACs effectively tested in the acute phase of VTE (i.e., in the first 5–10 days after the index event), while edoxaban may be used only after 5–10 days of LMWH (Fig. 2) [22–24].

While a minimum treatment period of 3–6 months for both LMWH and/or DOACs is recommended by current guidelines [7, 8, 11, 18–20], an extended indication for anticoagulation (beyond 6 months) should be carefully considered in selected cases, in particular in those patients with still active cancer, balancing bleeding and thrombotic risks [7, 8, 11, 18–20]. In this context, the recent EVE randomized controlled trial evaluated a dose reduction from 5 mg to 2.5 mg apixaban twice daily beyond 6 months of anticoagulation therapy in patients with cancer-associated VTE; among the 360 patients included in the intention-to-treat analysis, similar rates of major (hazard ratio [HR], 1.26; 95% CI, 0.34–4.66; *p* = 0.73) and clinically relevant non-major bleeding events (HR, 0.72; 95% CI, 0.38–1.37; *p* = 0.39) were observed with the 2.5 mg dose, without a substantial increase in terms of recurrent VTE, arterial thrombosis and all-cause mortality [30]. Analogously, in an open-label, adjudicator-blinded, randomized clinical trial, involving 32 institutions in Japan, and 178 patients with acute low-risk PE, 18-month rivaroxaban treatment (15 mg twice daily for the first 3 weeks followed by 15 mg once daily thereafter) was superior to a 6-month rivaroxaban regimen with respect to recurrent VTE events [31].

The recently published APIxaban for Cancer Associated Thrombosis (API-CAT) trial [32], enrolling 1766 patients with active cancer who completed at least 6 months of anticoagulant therapy for treating symptomatic or incidental VTE, showed that 2.5 mg twice daily apixaban is noninferior to 5 mg twice daily dose for the prevention of recurrent thrombotic events. Median treatment duration was 11.8 months (interquartile range, 8.3 to 12.1), during which recurrent VTE occurred in 18 patients (cumulative incidence, 2.1%) in the reduced-dose group, and in 24 (cumulative incidence, 2.8%) in the full-dose group (adjusted sub hazard ratio, 0.76; 95% CI, 0.41 to 1.41; *p* = 0.001 for noninferiority). Clinically relevant bleeding occurred in 102 patients (cumulative incidence, 12.1%) in the reduced-dose group, and in 136 (cumulative incidence, 15.6%) in the full-dose group (adjusted sub-hazard ratio, 0.75; 95% CI, 0.58 to 0.97; *p* = 0.03).

### Residual challenges with current anticoagulation strategies for cancer-associated venous thromboembolism

Anticoagulant management of VTE in subjects with active cancer is challenging as this population generally exhibits concomitantly higher risk for both recurrent VTE and bleeding events when compared with non-cancer patients [6, 33–35]. While anticoagulation is crucial for preventing potentially fatal sequelae, including massive PE, anticoagulant-related major bleeding events are generally more likely to result in death than thromboembolic recurrences [36, 37]. Hence, physicians taking care of patients with cancer-associated VTE often face complex therapeutic scenarios, including selection of the anticoagulation strategy, individual agent and dosing, as well as treatment duration and timing for treatment interruption/resumption in case of surgical procedures [38, 39]. Of importance, clinical decisions might be further complicated by the occurrence of active bleeding minor -bleeding) from intraluminal tumor masses or metastases, often located in critical sites (i.e., brain metastasis), thrombocytopenia, and liver or kidney dysfunction, which not unfrequently co-occur in subjects with active cancer [38, 39]. Patient- and disease-specific factors to consider include: cancer characteristics with location, histology and cytogenetics; the overall patient’s bleeding-thrombotic risk balance; comorbid conditions, often related to cancer disease itself or cancer therapy-induced toxicities, encompassing blood dyscrasias (e.g., anemia, thrombocytopenia) and liver/kidney dysfunction; drug-to-drug interactions (DDIs) between anticoagulants and other co-administered medications; patient performance status and, importantly, patient preferences and expectations [38, 39]. Therefore, adequate patient education and awareness throughout the cancer journey, in parallel with individualized multidisciplinary approaches to anticoagulant management, should be carefully considered [6, 40].

LMWHs, such as dalteparin and enoxaparin, have historically constituted the pillar of CAT treatment, carrying lower risks for DDIs/food-to-drug interactions in relation to VKAs, and not relying on gastrointestinal absorption (often unpredictable in cancer patients due to diarrhea, vomiting or malnutrition) [41]. As well, LMWHs do not require periodic international normalized ratio monitoring. Nevertheless, prolonged use of LMWHs may present drawbacks, including the requirement for subcutaneous, once- or twice-daily injections, which can be associated with pain and injection-site reactions, and may limit therapeutic adherence, particularly in subjects with advanced or end-stage malignancy, or in those requiring extended-duration anticoagulation. Additionally, the elevated cost and often limited availability of LMWHs may further limit accessibility to these agents [11, 38, 39].

DOACs nowadays represent the preferred anticoagulants in the majority of patients with CAT, with the exception of subjects considered at particularly high bleeding risk, especially those with unresected intraluminal malignancies, such gastrointestinal or genitourinary tumors [7, 8, 11, 18–20, 33]. It is also important to note that some differences in the safety profiles of individual DOACs have been suggested by more recent and larger analyses, incorporating real-life observational data, although no randomized controlled trial directly comparing two or more DOACs in this patient population has been conducted to date [42–44]. Overall, DOACs have contributed to a clinically meaningful reductions in VTE recurrence risk, and offer different practical advantages over LMWHs, including the oral route of administration, and the use at fixed rather than weight-adjusted dose [44]. Nevertheless, bleeding risk associated with DOACs remains an important concern. Thus, reducing and predicting such risk represents a major urgent unmet need in the contemporary VTE care [38, 39].

Furthermore, DOACs are metabolized in the liver via cytochrome P450 3A4 (CYP3A4) and their absorption/excretion is influenced by the P-glycoprotein (P-gp), and medications (e.g., certain chemotherapy agents, antibiotics and antifungals) that interfere with these pathways and may potentially lead to clinically relevant DDIs by altering the bioavailability and anticoagulant activity of DOACs, thus affecting their safety and efficacy profiles [45, 46]. Of importance, these concerns should be reshaped in the frame of a recent research by Truong et al., analyzing adverse event reports including DOACs and antineoplastic agents with CYP3A4/P-gp inhibitory/inducing activity from the US Food and Drug Administration Adverse Event Reporting System [47]. These data showed no signal of DDIs between DOACs and antineoplastic agents, as well as most reported DDIs appeared to be non-clinically relevant [46].

Both liver and kidney diseases are relatively frequent among patients with cancer. Because of their hepatic metabolism, DOACs are contraindicated in subjects with severe liver disease (Child-Pugh C) [48]. Similarly, since DOACs excretion largely relies on renal clearance, kidney dysfunction can lead to drug accumulation and increased bleeding risk. Caution should be therefore paid in subjects with chronic kidney disease and moderately-to-severely impaired creatinine clearance (i.e., 30 to 50 ml/min). As well, DOACs are contraindicated in those subjects with advanced renal impairment (creatinine clearance < 15 mL/min for edoxaban, apixaban and rivaroxaban, or creatinine clearance < 30 mL/min for dabigatran) or undergoing dialysis [48]; at the moment, no clinical evidence related to the safety of DOACs is available for the treatment of VTE in patients with severe renal impairment, while real-word data are accumulating [48].

Oncologic patients may face percutaneous endoscopic gastrostomy (PEG) or feeding tubes positioning, as well as extended intestinal resections, which obstacle DOACs bioavailability and question an optimal way of administration. Intuitively, patients with significantly altered gastrointestinal tracts were not included in clinical trials assessing DOACs efficacy and safety [49]. In particular, specific considerations should be made in terms of concomitant food consumption (especially in the case of rivaroxaban), major gastrointestinal absorption site (e.g., stomach for edoxaban and rivaroxaban, while small bowel for apixaban and dabigatran) and possibility of crushed formulations [49]. So far, edoxaban alone has been tested in the setting of fragile patients with PEG and atrial fibrillation, showing safety in terms of MB and efficacy, as demonstrated by drug’s measured plasma concentrations [50]. These results may pave the way to dedicated research in the oncologic population.

Besides lower-extremity proximal DVT and PE, when compared with non-cancer population, oncologic patients also are at substantially higher risks to develop isolated distal DVT, subsegmental PE, and catheter-related thrombosis (the latter due to the frequent use of indwelling lines to administer intravenous cancer-directed therapies), whose treatment is recommended according to guidelines [11, 51–53].

Highly controversial appears the management of breakthrough thrombosis, that configures as uncommon but challenging events. It is common knowledge that in these cases, a switch from an anticoagulation regimen to another should be considered, although evidence available only refers to the pre-DOACs era, and advocates for an increase in LMWH dose increase [54].

Frailty and comorbidities related to cancer patients should be considered with caution when high-risk PE occurs. In non-cancer patients, both invasive and non-invasive options are available, in particular in cases of hemodynamic instability [55]. In particular, the use of systemic thrombolytics has been shown to lead to faster improvements of pulmonary obstruction and right ventricular dimensions, when compared to unfractionated heparin [55]. Plasminogen activator-based therapies, including recombinant tissue plasminogen activator (r-tPA/Alteplase) are however, burdened in clinical practice by unacceptable bleeding events, which may be even more frequent in patients with active malignancies [54]. Therefore, although scarce available evidence and high peri-procedural risk, mechanical reperfusion by catheter-directed fragmentation, thrombus aspiration, even combined with *in-situs* reduced-dose thrombolysis, may be considered as an alternative option for management of high-risk PE in this setting [54]. Inferior vena cava filters, which have shown an increased rate of PE-free survival [55], should be carefully considered for implantation, in particular in cases of acute venous thrombosis with an existing absolute contraindication to anticoagulation or patients recurrent VTE despite anticoagulant therapy [19, 55, 56].

### New therapeutic strategies for cancer-associated venous thromboembolism

#### From hemostasis to thrombosis and the key factor XI, with a focus on patients with cancer

**Fig. 3:**
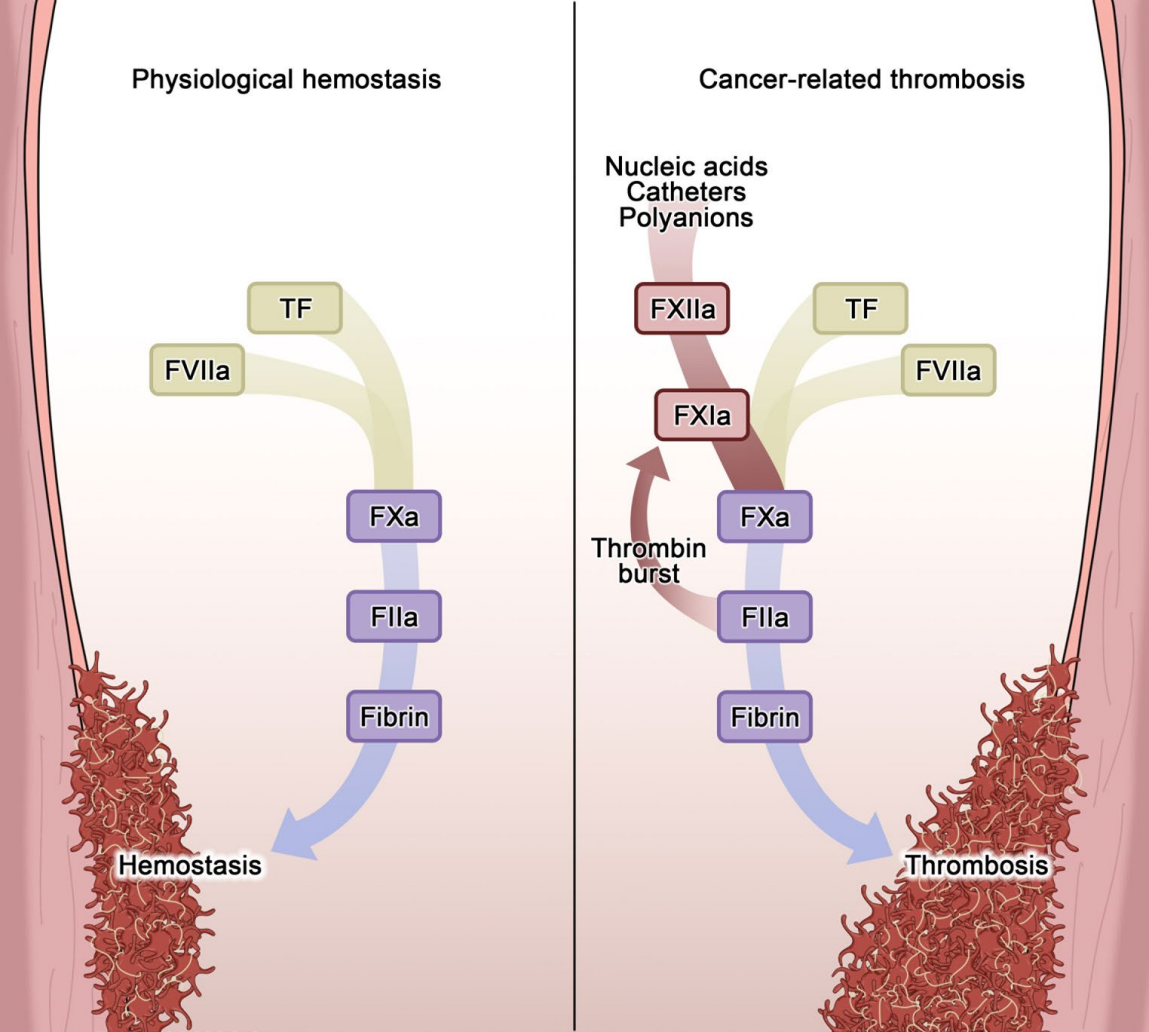
Mechanisms behind physiological hemostasis and cancer-related thrombosis. The figure displays the pathophysiological mechanisms of hemostasis (left) and cancer-related thrombosis (right). During physiological hemostasis, the pronounced release of TF from damaged tissues makes the direct pathway of coagulation (yellow stream) the main driver of the process, culminating in the activation of the common pathway (blue arrow). Conversely, in cancer-related thrombosis, the indirect pathway (red stream) is more strongly activated due to the presence of long-term catheters and negative-rich molecules released from the damaged tissues, with additive effects on the physiological hemostatic process. The continuous activation of factor XI from FIIa ultimately leads to a continuous loop of activation and continuous generation of FIIa, also known as “thrombin burst”, with FXIa being the milestone of the loop. Abbreviations: FIIa, Thrombin; FVIIa, Activated factor VII; FXa, Activated factor X; FXIa, Activated factor XI; FXIIa, Activated factor XII; TF, Tissue factor

Before speculating on the application of Factor XI inhibitors in the management of CAT, a brief introduction on the role of Factor XI in the hemostatic process appears warranted (Fig. 3).

Coagulation is a deeply intricated cascade of multiple enzymes and cells, leading to hemostasis in physiologic conditions or pathological arterial and venous thrombosis [57]. The coagulation process consists of three major steps, namely initiation, amplification and consolidation [57]. In physiological hemostasis, during initiation phase, tissue damage triggers the extrinsic pathway through the interaction of circulating factor VII (FVII) and tissue factor (TF), expressed by local cells. This pathway converges into the amplification phase, where sustained activation of factors IX and X generates a large amount of thrombin. Finally, during the consolidation phase, thrombin activates factor XI (FXI), contributing to sustained thrombin generation, thus consolidating the hemostatic plug. However, the consolidation phase, involving FXI, plays a limited role in hemostasis, as evidenced in individuals with congenital FXI deficiency (hemophilia C), who experience generally milder bleeding events compared to other coagulation factor deficiencies [58, 59].

In contrast, in pathological arterial and venous thrombosis, thrombus growth away from the vessel wall after extrinsic pathway activation, heavily relies on FXI to amplify the process (i.e., amplification phase), as the TF-FVIIa complex alone may be insufficient once the thrombus extends beyond the TF source [57]. The key role of FXI has been highlighted in Mendelian randomization studies, where high FXI levels were associated with an increased risk of VTE and ischemic stroke [60]. Conversely, lower FXI levels correlate with reduced risks of both VTE and ischemic stroke, without an increased risk of major bleeding [61].

As for the hemostatic process, thrombin is the major FXI activator in pathological arterial and venous thrombosis [57]. Nevertheless, FXI activation may also occur via factor XII (FXII), as a consequence of blood exposure to artificial or negatively-charged surfaces, a process particularly enhanced in cancer patients. With regards to the extrinsic pathway activation, certain cancer cell types constitutively express TF and release extracellular vesicles bearing TF, while monocytes in cancer patients also exhibit increased TF expression [62, 63] (see also Fig. 1). Regarding intrinsic pathway activation, FXI activation via FXIIa is particularly enhanced in cancer patients, promoted by the presence of artificial surfaces, such as central venous catheters [64]. In addition, local mediators released by cancer cells (i.e., plasmin, collagenase, cathepsin, polyphosphates, glycosaminoglycans, cell-free DNA, neutrophil extracellular traps, and carcinoma mucins) degrade extracellular components, leading to negatively-charged surfaces exposition and stimulating FXI activation by FXIIa [65–67]. Finally, tumor-derived microvesicles, enriched with TF, phospholipids and long-chain polyphosphates, have been implicated in the hypercoagulable state leading to FXII-related FXI activation associated with malignancies [62, 68].

#### Pharmacological inhibition of factor XI: implications for cancer-associated thrombosis

In this scenario, therapeutic strategies that selectively target FXI have emerged as a promising alternative to traditional anticoagulant approaches (Table 3) [69]. By attenuating thrombus formation while preserving primary hemostasis, FXI inhibition may have the potential to redefine the anticoagulation landscape, especially in patients with cancer, mitigating thrombotic risk without incurring an unacceptable bleeding diathesis. Conversely, although data from animal models have shown that FXII deficiency or inhibition attenuates arterial and venous thrombosis, epidemiologic studies failed to demonstrate protection in subjects with FXII deficiency and reported an inconsistent association between higher FXII levels and thrombotic risk [70, 71]. Administration of FXI inhibitors to prevent thrombotic events was initially explored in trials of total knee arthroplasty, which consistently showed that FXI inhibitors are superior to enoxaparin in reducing post-operative thromboembolism, maintaining a comparable bleeding profile [72]. Data on patients with cancer are still pending, with abelacimab and gruticibart being the only molecules currently under evaluation in this setting.

**Table 3:** Pharmacological features of factor XI-directed strategies

	Monoclonal antibodies (abelacimab, gruticibart, osocimab)	Antisense oligonucleotides (fesomersen)	Small molecules (asundexian, milvexian)
Mechanism of action	Bind target protein	Block synthesis	Bind target protein
Administration	Intravenous or subcutaneous	Subcutaneous	Oral
Onset of action	Rapid (hours to days)	Slow (weeks)	Rapid (1 to 4 h)
Offset of action	Slow (weeks)	Slow (weeks)	Rapid (12 to 24 h)
Renal clearance	No	No	Minimal
Potential for drug-drug interactions	No	No	Yes *(except for asundexian)*
CYP450 metabolism	No	No	Yes *(except for asundexian)*
Under evaluation or preliminarily evaluated in cancer patients	Yes *(abelacimab*,* gruticibart)*	No	No

Abelacimab (MAA868) is a fully-human monoclonal antibody able to inhibit both FXI and FXIa by interaction with the catalytic domain [73]. Abelacimab is associated with prolonged and sustained dose-dependent prolongation of activated partial thromboplastin time (aPTT), without significantly influencing prothrombin time (PT). Of importance, its particularly long half-life of about 25–30 days allows monthly administration [74]. In addition, its clearance primarily involves the reticuloendothelial system, rather than renal or hepatic metabolism, thus having low potential DDIs and not being influenced by organ dysfunction. Nevertheless, as a drawback, abelacimab lacks of a specific antidote. However, strategies to prevent or treat bleeding include the use of tranexamic acid, low-dose FVIIa, or activated prothrombin complex concentrates [75]. The ASTER and MAGNOLIA trials are the two ongoing studies, for which a fast-track designation has been granted by the Food and Drug Administration in 2022.

The ASTER trial (NCT05171049) is currently exploring the non-inferiority of intravenous abelacimab 150 mg compared to apixaban (10 mg twice daily for seven days, followed by 5 mg twice daily) in reducing 6-months VTE recurrences in patients with a diagnosis of cancer and symptomatic or incidental proximal DVT or PE. Secondary endpoints include the assessment of major and clinically relevant non-major bleeding events, as well as the evaluation of net clinical benefit, defined as survival without VTE recurrence or major bleeding events at 6 months. The MAGNOLIA trial (NCT05171075) is comparing intravenous abelacimab 150 mg with dalteparin (200 IU/kg/day for the first month, followed by 150 IU/kg/day) in DOACs-ineligible patients with advanced gastrointestinal or genitourinary cancer and symptomatic or incidental VTE. The primary efficacy endpoint is time to the first event of centrally-adjudicated VTE recurrence, while secondary endpoints include time to the first event of major or clinically relevant nonmajor bleeding events, and net clinical benefit at 6 months.

FXI inhibition through administration of xisomab 3G3 (AB023, gruticibart) has been recently explored as strategy to prevent catheter-associated thrombosis [76]. Xisomab 3G3 is a recombinant monoclonal antibody being able to target FXIIa-dependent activation of FXI, thus preventing thrombosis [77]. Two phase 2, single-center studies (NCT04465760) were conducted in 22 ambulatory cancer patients who underwent central line placement (subclavian central venous catheter and double lumen peripherally inserted central venous catheter) for chemotherapy initiation. Patients received a single dose (2 mg/kg, through the catheter within 24 h from placement) of xisomab 3G3, and underwent ultrasound evaluation after 14 days. Compared to the control group, in patients in the investigational arm, a lower incidence of catheter-associated thrombosis was reported (12.5% vs. 40.0%), along with a significant prolongation of the aPTT and no differences in platelet activation among groups. Finally, the molecule was well-tolerated with no adverse or bleeding event.

In aggregate, administration of FXI inhibitors in cancer patients is sustained by a biological plausibility, having the promise to significantly improve overall net-benefit in this population that is at higher risk of bleeding. In particular, molecules such as abelacimab, characterized by long half-life and no DDIs, may represent a changing-paradigm treatment, although there is currently lack of high-quality evidence from large-scale, randomized clinical trials [78]. The results of the ASTER and MAGNOLIA trials, expected in 2026, will be critical in determining the safety and efficacy of a FXI inhibitor in CAT, and may prompt further exploration with other molecules in the field.

#### Other horizons for management of thromboembolism

As mentioned above, plasminogen activator-based thrombolytic therapies were approved for VTE treatment, while fatal bleeding events were registered in clinical practice [76]. For this reason, this therapeutic approach is almost reserved to patients with high-risk PE with hemodynamic instability. In this context, the ongoing PEITHO (Pulmonary Embolism International THrOmbolysis, NCT04430569) trial is investigating safety and efficacy outcomes of low dose r-tPA (0.6 mg/kg ≤ 50 mg) in non-cancer patients with intermediate-high-risk PE at 30 days, along with long-term follow-up [78, 79].

In addition, the notion that considers that venous thrombi can be dissolved without significantly increasing the bleeding risk by targeting fibrinolysis inhibitors, such as alpha2-antiplasmin (alpha2AP), plasminogen activator inhibitor-1 (PAI-1), and thrombin activable fibrinolysis inhibitor (TAFI), is now emerging in the non-cancer population [80, 81]. Antagonists of fibrinolytic inhibitors directly target thrombus resistance and accelerate thrombus dissolution, either independently or in combination with several-fold lower doses of r-tPA [78]. Pharmacologic inhibitors directed against these molecules have been mainly tested in the preclinical setting, while very few phase 2 clinical studies are now investigating their efficacy in non-oncologic subjects with acute sub-massive PE or proximal DVT [77]. Therefore, more research insights are needed into the pathogenesis of VTE in cancer patients, in order to enhance the design of dedicated randomized trials with these novel compounds. From a translational perspective, anti-polyphosphate agents, anti-factor XII antibodies, anti-myeloperoxidase (MPO) agents and anti-P-selectin antibodies are all aimed at interfering with the coagulation and inflammatory cascade. Anti-polyphosphate agents target polyphosphate, a key factor in blood clot formation; anti-FXII antibodies target Factor XII of the coagulation process, while anti-MPO agents and anti-P-sel antibodies target myeloperoxidase and P-selectin, which are involved in inflammation, neutrophil activation and NETosis. As preliminary demonstrated in pre-clinical studies, targeting these molecules holds promise in preventing thrombosis in patients with cancer without bleeding risk increase.

At the same time, antiplatelet agents, including aspirin and/or antagonists of cell-surface receptors of platelets including PAR1, P2Y12, and αIIbβ3, have raised attention in the setting of thrombosis prevention, with mixed results [78, 82, 83]. The application of preventive strategies beyond anticoagulants should to be tested in the cancer patients setting.

Even in this field of CAT, artificial intelligence may help transforming Cardio-Oncology practice, enabling personalized care for oncologic patients at highest risk of thrombotic and/or bleeding risk [84].

## Conclusions

Despite the advancesintroduced by DOACs, and with the promise of factor XIa inhibition approaching the horizon of CAT, current therapeutic management of this condition still harbors significant challenges that are frequently faced by patients and healthcare professionals in complex clinical decision making.

Abelacimab holds promise for reducing bleeding risk and overcoming potential DDIs as well as concerns about kidney or hepatic dysfunction. However, the safety and efficacy of pharmacological inhibitors of factor XI in this highly vulnerable population still need to be evaluated in ongoing randomized controlled trials. In addition, the simultaneous therapeutic development of different classes of XIa inhibitors (monoclonal antibodies, oral small molecule inhibitors, and antisense oligos) may represent a multifaceted approach to address the patient-management needs such as parenteral vs. oral administration, or immediate vs extended inhibition in different clinical subsets.

## Electronic supplementary material

Below is the link to the electronic supplementary material.

Supplementary Material 1

## Data Availability

No datasets were generated or analysed during the current study.
