# Supplementary material for: Management of cancer-associated venous thromboembolism: Perspectives on optimizing current therapeutics with a focus on factor XI inhibition
Source: J Thromb Thrombolysis. 2025 Jul 29;58(8):1095–108. doi: 10.1007/s11239-025-03154-7 (PMC12740953; doi:10.1007/s11239-025-03154-7)
Supplement: Supplementary file 1 — Supplementary Material 1 [file 11239_2025_3154_MOESM1_ESM.docx]

Table S4. Complete list of search terms and results.

| **Set#** | **Searched for** |
| --- | --- |
| S1 | Venous thromboembolism OR VTE OR pulmonary embolism OR deep vein thrombosis OR DVT OR venous thrombosis OR cancer-associated thrombosis OR CAT |
| S2 | cancer OR oncology OR neoplasm OR tumour OR malignancy OR carcinoma OR metastasis |
| S3 | Direct oral anticoagulant OR DOAC OR Anticoagulant OR Novel oral anticoagulants OR NOAC apixaban OR Eliquis OR edoxaban OR Lixiana OR rivaroxaban OR Xarelto OR dabigatran OR Pradaxa OR "oral Xa inhibitor |
| S4 | Heparin OR Unfractionated heparin OR UFH OR Low-molecular weight heparin OR LMWH OR dalteparin OR Fragmin OR Boxol OR fondaparinux OR Arixtra OR Xantidar OR fondaparin |
| S5 | Factor XI inhibitor OR Abelacimab OR MAA868 OR Gruticibart OR AB023 OR Xisomab3G3 OR Osocimab OR BAY1213790 OR Fesomersen OR IONIS-FXI-LRx OR Asundexian OR BAY2433334 OR Milvexian OR BMS-986177 OR JNJ-70033093 |
